# Hodgkin Lymphoma Presenting With Spinal Cord Compression: Challenges for Diagnosis and Initial Management

**DOI:** 10.1177/10935266211033269

**Published:** 2021-08-24

**Authors:** Nicola Bloxham, Justin Cross, Matthew Garnett, Jessica Bewick, Kate Armon, C Elizabeth Hook, Matthew J Murray

**Affiliations:** 1 Department of Paediatric Haematology and Oncology, 2153 Cambridge University Hospitals NHS Foundation Trust, Cambridge University Hospitals NHS Foundation Trust, Cambridge, UK; 2 Department of Radiology, 2153 Cambridge University Hospitals NHS Foundation Trust, Cambridge University Hospitals NHS Foundation Trust, Cambridge, UK; 3 Department of Neurosurgery, 2153 Cambridge University Hospitals NHS Foundation Trust, Cambridge University Hospitals NHS Foundation Trust, Cambridge, UK; 4 Department of Ear, Nose and Throat Surgery, 2153 Cambridge University Hospitals NHS Foundation Trust, Cambridge University Hospitals NHS Foundation Trust, Cambridge, UK; 5 Department of Paediatric Rheumatology, 2153 Cambridge University Hospitals NHS Foundation Trust, Cambridge University Hospitals NHS Foundation Trust, Cambridge, UK; 6 Department of Paediatric Histopathology, 2153 Cambridge University Hospitals NHS Foundation Trust, Cambridge University Hospitals NHS Foundation Trust, Cambridge, UK; 7 Department of Pathology, 2152 University of Cambridge, University of Cambridge, Tennis Court Road, Cambridge, UK

**Keywords:** cord compression, diagnosis, Hodgkin, management, spinal cord

## Abstract

Hodgkin lymphoma (HL) can present with extra-nodal disease, but spinal cord compression is exceptionally rare. We describe a 15-year-old presenting with hip/back pain with normal initial examination. Persistent pain and raised inflammatory markers prompted further investigation with MRI, which revealed an epidural mass causing spinal cord compression. On examination, there was no palpable lymphadenopathy or cauda equina syndrome, but absent lower limb reflexes were noted. Following multidisciplinary discussion, it was determined that cauda equina syndrome was imminent and therefore surgical debulking was undertaken, both to prevent this complication and establish a diagnosis. At surgery, the tumor was highly vascular. Frozen section confirmed lesional material. Following surgery, and given the frozen section findings, a short course of steroids was commenced to reduce any peri-surgical edema. Unfortunately, histopathology was ultimately non-diagnostic, due to failure of immunohistochemistry on technically challenging material. Consequently, ultrasound-guided excision biopsy of a (non-palpable) cervical lymph node was performed five days later; histopathology showed typical effacement of the normal architecture and a conspicuous population of CD15/CD30-positive larger pale cells present, confirming nodular sclerosis classic HL, despite recent steroids. We review the available literature for HL presenting with spinal cord compression and describe the challenges for diagnosis and initial management in such cases.

## Introduction

Hodgkin lymphoma (HL) is primarily a malignancy of the lymph nodes but can present with extra-nodal involvement. Incidence follows a bimodal age distribution, rising sharply during childhood and peaking initially in young adults aged 20-24 years (y), with a second larger peak in incidence during the eighth decade of life. In the UK, 435 children and young adults (<25y of age) are diagnosed with HL each year,^1^ with an average incidence rate of 9.4 per 100,000 for those 20-24y.^1^ HL is characterised by the presence of binucleated giant cells termed Reed-Sternberg cells or large mononuclear cell variants (lymphocytic and histiocytic cells) on a background of inflammatory cells and is broadly divided into two pathologic classes: classic or nodular lymphocyte predominant disease.^2,3^ Immunophenotyping is essential to distinguish between the two classes, with classic HL expressing CD30 in nearly all cases and CD15 in 75-85%, whilst typically lacking expression of the B-cell markers (CD19, CD20 and CD79a).^3,4^ PAX-5 is the only B-cell restricted antigen that is nearly always expressed in classic HL, but staining is often weaker than that seen in reactive B-cells and is a useful diagnostic feature.^4^ Classic HL accounts for the majority of childhood, adolescent and young adult cases,^2,3^ with the nodular sclerosis type being the most common subtype, primarily affecting adolescents and young adults (15-34y) with a female predominance.^3–5^ Treatment advances have dramatically increased HL survival over the past 40 years across all patient age-groups. Age-standardised 10y survival has increased from 47% in 1971 to 80% in 2011 in the UK.^1^ Outcomes are better still for children and young adults, with 5y survival exceeding 95% across much of Europe and the US.^1,2,5^ Current trials in this group are aimed at maintaining overall survival whilst minimising the morbidity associated with the late-effects of treatment, with a move away from radiotherapy as standard treatment.^2,6^

A prolonged time-to-diagnosis is recognised in patients with cancer, including HL,^7–9^ which may result in more advanced stage at presentation.^1^ Although overall survival is generally not compromised in such cases, advanced stage disease requires more treatment for cure and hence late-effects are likely to be more substantial. Consequently, raising awareness of such extra-nodal cases is important to minimise time-to-diagnosis and late sequelae. In particular, extra-nodal HL only very rarely presents with spinal cord compression (SCC), described in only a few rare case reports.^10–12^ Hence, it may not be in the list of potential differential diagnoses and management of such patients may be suboptimal as a result. Here, we describe the case of a pediatric, adolescent patient presenting with SCC and discuss the challenges for HL diagnosis and initial management.

## Case Report

A 15-year-old female presented to primary care after 2 months (68 days) of left hip pain and shooting pains in the left shin. She was given non-steroidal anti-inflammatory drugs with some improvement, and referred to pediatric rheumatology after blood testing revealed raised inflammatory markers [high erythrocyte sedimentation rate (ESR) 76mm/hr and C-reactive protein (CRP) 17mg/L]. Full blood count (FBC) at this time revealed hemoglobin 127g/L, white cell count 10.7 × 10^9^/L, and platelets 444 × 10^9^/L. Urea and electrolytes (U&E) and bone (BFT) and liver function tests (LFT) were normal and antinuclear antibody (ANA) and rheumatoid factor were negative. At four months from symptom onset (117 days), she was seen in pediatric rheumatology, where clinical examination was normal, and further investigations revealed persistently elevated CRP (44mg/L) and ESR (75mm/hr); other bloods including repeat FBC, U&E, LFTs, creatine kinase (CK) and immunoglobulins were all normal. Five months after symptoms onset (159 days) left hip pain was worse, and lower back pain and stiffness had developed causing antalgic gait. The MRI of hip, spine and pelvis was delayed due to the COVID-19 pandemic, and undertaken seven months after symptom onset (208 days), showing an epidural soft tissue mass, resulting in referral to pediatric oncology for suspected cancer.

**Figure 1.**
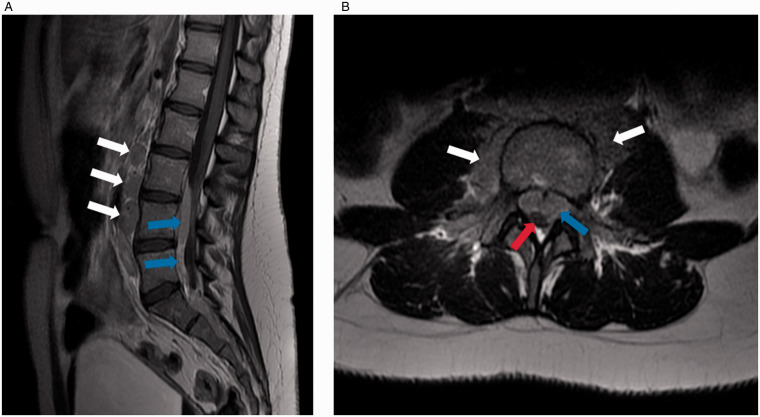
Representative MRI imaging of the epidural mass causing spinal cord compression. A, Sagittal T1 weighted contrast imaging demonstrating the anterior epidural enhancing mass in the lumbar canal (blue arrows) with enlarged paraaortic lymph nodes (white arrows). B, Axial T2 weighted imaging demonstrating enlarged paraaortic lymph nodes and abnormal paraspinal soft tissue (white arrows) and the anterior epidural mass (blue arrow). The thecal sac (red arrow) is displaced posteriorly and compressed.

By this time, examination findings were bilaterally absent lower limb reflexes only, with no clinical evidence of cauda equina syndrome. No palpable lymphadenopathy was detected. The MRI scan demonstrated widespread lymphadenopathy, with multiple enlarged carotid and submandibular lymph nodes as well as numerous para-aortic lymph nodes extending around the fourth lumbar (L4) vertebral body and via the L3/4 and L4/5 neural foramina bilaterally into a largely anterior epidural mass (Figure 1(A)). The mass extended inferiorly, occupying most of the sacral canal and extended anteriorly through the sacral foramina. The thecal sac was displaced and moderately compressed at the lumbar region below L3 with enhancement of the cauda equina and severe thecal compression in the sacrum (Figure 1(B)). Radiologically, the most likely differential diagnosis was lymphoma.

**Figure 2.**
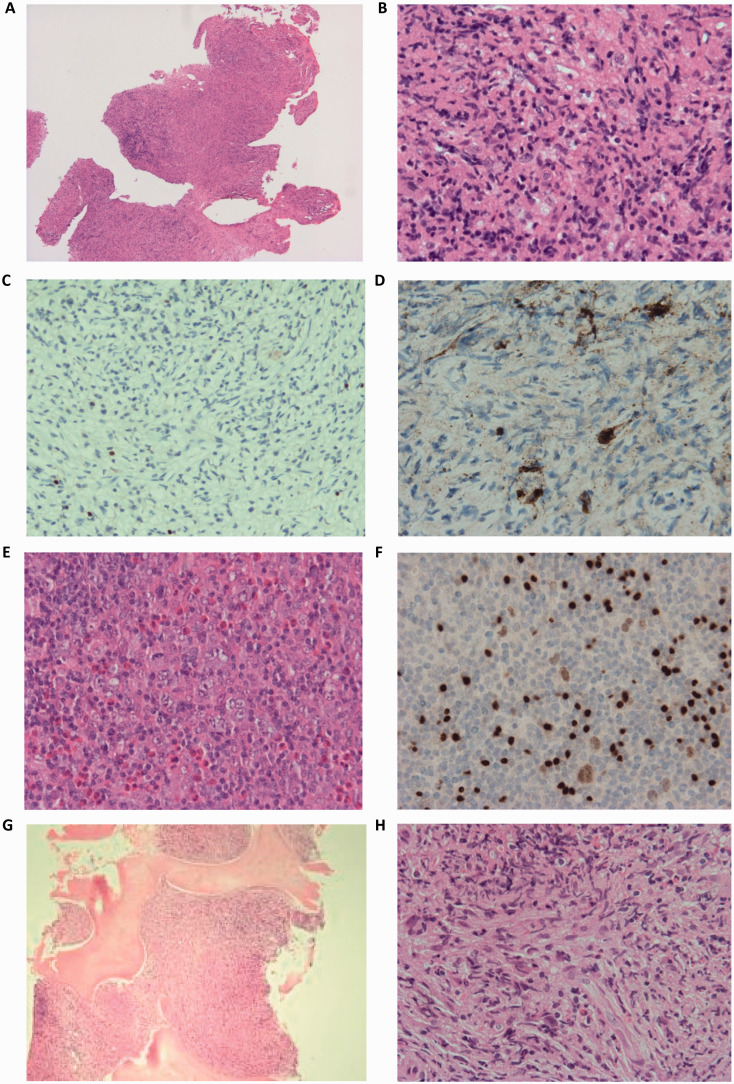
Representative pathologic findings in the spinal cord compression Hodgkin lymphoma case. Original (epidural) biopsy images showing (A) architecture (x40 magnification), (B) eosinophils and atypical cells (x400) and immunohistochemistry: (C) negative PAX-5 staining (x200) and (D) positive CD30 staining (x400). Images of the second (right cervical lymph node) biopsy revealing (E) atypical large cells and eosinophils (x400) and (F) large, atypical pale cells with PAX-5 staining (x400). Staging bone marrow images showing (G) replacement of the normal bone marrow with a malignant infiltrate (x40) and (H) eosinophils and atypical cells (x400). These atypical cells were positive for CD30 staining, consistent with stage 4 Hodgkin lymphoma.

Following multidisciplinary discussion, it was determined that cauda equina syndrome was imminent and therefore surgical debulking was undertaken, both to prevent this complication and establish a diagnosis, with emergency decompression of the theca and left L4 nerve root. At surgery, the tumor was noted to be highly vascular. Frozen section confirmed lesional material. Following surgery, a short course of oral steroids was commenced to reduce any peri-surgical edema. However, the material presented for histopathologic examination was ultimately non-diagnostic, both by conventional pathology and urgent flow cytometry. Samples showed poorly preserved connective tissue with diathermy and freezing artefacts. An excess of eosinophils was seen with some larger cells noted (Figure 2(A) and (B)). Immunostaining of these larger, atypical cells showed CD30 and very weak MUM-1 expression, but overall did not confirm a diagnosis of HL as expression of CD15 and PAX-5 was not seen (Figure 2(C) and 2(D)) and morphology was not typical. Flow cytometry was non-contributory. Further immunostains, including negative CD1a (excluding Langerhans Cell Histiocytosis) and ALK (excluding Anaplastic Large Cell Lymphoma) were performed, but with concerns noted over the suitability of the tissue for such immunostaining given the diathermy and freezing artefacts. All available lesional material was used for these analyses but unfortunately a diagnosis was not secured.

Consequently, and following further MDT discussion, open surgical excisional biopsy of (non-palpable) cervical lymph nodes was performed five days later as guided by ultrasound findings, along with bone marrow sampling due to the putative HL diagnosis, and insertion of a central venous access device in order to deliver systemic chemotherapy. On histopathology, effacement of the normal lymph node architecture was observed, with prominent bands of fibrosis and a marked infiltrate of eosinophils. A conspicuous population of larger pale cells was present with varying nuclear morphology (Figure 2(E)). These larger, atypical cells stained for CD15 and CD30 with weak PAX-5 positivity (Figure 2(G) and (H)). Immunostains for BCL-6, CD79a, CD20, CD45 and ALK were all negative. The findings were consistent with nodular sclerosis classic HL. Bone marrow trephines demonstrated the presence of atypical cells and an excess of eosinophils, confirming the presence of (stage 4) metastatic disease (Figure 2(G) or (H)). A staging ^18^fluoro-deoxyglucose (FDG) positron-emission-tomography (PET) scan demonstrated high tracer uptake in enlarged lymph nodes on both sides of the diaphragm, spleen, retroperitoneal nodules, paraspinal soft tissue and bone marrow, consistent with stage 4 disease. During the final few days of diagnostic work-up, prior to commencement of treatment, the patient developed ‘B’ symptoms (night sweats) and therefore assigned 4B staging. The patient was enrolled on the EuroNet-PHL-C2 Phase 3 trial for children and young adults (<25y) with classic HL, in treatment level 3 (TL3), and responded well to treatment. Early-response-assessment FDG-PET after two cycles of induction ‘OEPA’ chemotherapy confirmed the need for radiotherapy following completion of chemotherapy. Late-response-assessment FDG-PET after four cycles of consolidation ‘COPDAC’ chemotherapy showed further treatment response but showed some residual increased uptake at the site of disease around the L4 vertebral body, confirming the need for radiotherapy boost to this area.

## Discussion

This case highlights a number of challenges in establishing the diagnosis, and of the early management of spinal cord compression (SCC), in the context of previously undiagnosed Hodgkin lymphoma (HL). Prompt diagnosis of HL is important to minimise the morbidity associated with the late-effects of the more intensive treatment required to cure high-stage disease, and, in the setting of SCC, to optimise functional outcomes. Whilst we are not aware of published data on the time-to-diagnosis of HL in purely pediatric populations, a recent published study found that self-reported time-to-diagnosis was a median of 158 days (interquartile range 84-288 days) for HL in an adult population (>18y).^8^ Of this time-to-diagnosis, the median diagnostic interval (help-seeking to diagnosis) was nearly three times greater than the median patient interval (symptom onset to help-seeking), namely 87 days and 30 days, respectively.^8^ These findings are consistent with this case, with a protracted diagnostic interval and total time-to-diagnosis of seven months, exacerbated by the COVID-19 pandemic, and similar to other reports.^13^ As a result of variability in time-to-diagnosis, over one-quarter of HL patients present with advanced stage (3 or 4) disease.^1^ The majority (80%) of patients with classic HL present with painless lymphadenopathy, usually involving the supraclavicular and cervical nodes.^2,4^ Of note, our patient did not have palpable lymphadenopathy, adding to the diagnostic challenge. A similar proportion of adolescents and young adults will have anterior mediastinal involvement at presentation, often asymptomatic.^2,4^ Extra-nodal involvement usually arises from hematogenous dissemination.^4^

SCC is a rare extra-nodal manifestation of HL, occurring in ∼5% of cases and usually in the context of progressive or advanced disease.^11,12^ It is the main presenting feature in only ∼0.2% of cases.^10^ Epidural lesions in HL may arise from hematogenous dissemination but are more likely to develop as a result of local invasion from retroperitoneal or thoracic lymph nodes.^10^ The thoracic spine appears to be the most commonly implicated site, followed by the lumbar spine.^10^ Of note, back pain is not an uncommon complaint in the pediatric population, with a prevalence of ∼10% at age 10y and ∼30% at 13y of age.^14^ That notwithstanding, chronic back pain has a relatively high yield for serious pathology including mechanical, infectious, inflammatory and neoplastic etiologies. For this reason, it is important to keep a very broad differential when a child or adolescent presents with back pain. For example, back pain in a child causing waking from sleep should result in urgent referral to pediatrics^15^ and neurological deficits should also always be considered a ‘red flag’ and urgent imaging arranged. A study of patients presenting with a malignant cause for spinal cord compression found that neuroblastoma (29%), soft-tissue sarcomas (21%), neuroectodermal tumors (17%) and non-Hodgkin lymphoma (13%) made up the majority of cases.^16^ Other malignant causes included astrocytoma, Wilms tumor and leukemia.^16^ Another study showed that SCC was the presenting feature of a previously undiagnosed malignancy in 75% of cases.^17^ Motor deficit was the presenting symptom in all patients, while pain was reported in 60% and sphincter dysfunction in 43%.^17^

The UK NICE guidelines for the emergency management of malignant SCC recommend steroids as an essential component and that they should be offered to all patients while definitive treatment is planned.^18^ Steroids are, however, contraindicated in those patients with a significant suspicion of lymphoma.^18^ HL is highly steroid sensitive, with treatment protocols utilising them as a key backbone of therapy.^6,19^ Even very short courses of steroids prior to biopsy may reduce the likelihood of reaching a definitive and accurate histologic diagnosis in such cases. Although most cases of classic HL can be diagnosed on morphologic and immunophenotypic features, there are a number of malignant lymphoid proliferations that can display histological features resembling HL. These include grey-zone lymphoma, EBV-positive diffuse large B-cell lymphoma, anaplastic large-cell lymphoma and peripheral T-cell lymphoma with ‘Hodgkin-like’ cells.^4^ Despite steroids being withheld in this case for these reasons until after the first biopsy, a diagnosis could not be reached from the spinal biopsy alone. In this clinically emergent setting of spinal cord compression, a rapid and accurate pathological diagnosis is however important. It should be noted that although the morphological appearances of the original epidural biopsy were concerning for HL, and a very small minority of HL cases can be PAX5 negative, the combination of negative staining for both CD15 and PAX-5 in this first biopsy in conjunction with the poorly preserved morphology, meant it was not felt possible to make a formal diagnosis on this tissue. This is likely to be related to poor preservation of tissue due to technical (diathermy/freezing artefact) reasons; immunohistochemistry is sensitive to tissue handling and preservation. However, use of diathermy was required to safely neurosurgically decompress the spinal cord and nerve roots, given the highly vascular nature of the tumor. In this case, as the patient had undergone emergency decompression of the theca and left L4 nerve root and had started steroids, with some clinical improvement, and given the putative HL diagnosis, it was therefore deemed clinically prudent to obtain more tissue to securely establish the exact diagnosis under the same general anaesthetic used for central line placement and staging bone marrow examination five days later. Clearly, such a decision for repeat biopsy or otherwise has to be assessed on a case-by case basis. In certain instances, where repeat biopsy is not feasible, or if repeat biopsy is non-diagnostic due to steroid delivery, a pragmatic and empirical diagnosis may have to be accepted based on the findings of the original biopsy.

In summary, we present a 15-year-old with HL presenting with SCC, advanced stage disease and prolonged time-to-diagnosis. The diagnosis was challenging as the first neurosurgical biopsy was non-diagnostic, resulting in a second ultrasound-guided excision biopsy of a non-palpable cervical lymph node five days later, that allowed the diagnosis of nodular sclerosis classic HL to be made, despite the interim use of steroids following the first biopsy. The case highlights that pediatric and adolescent patients presenting with back pain need timely investigation and onward referral if any abnormal signs, symptoms or results, and the need to maintain a broad differential when assessing such patients. Such an approach will minimize diagnostic intervals and result in optimized patient outcomes.
